# Charge-dipole bound anion-anion complexes

**DOI:** 10.1007/s00894-026-06947-6

**Published:** 2026-09-15

**Authors:** Jane S. Murray, Timothy Clark

**Affiliations:** 1 Department of Chemistry, LSU New Orleans, New Orleans, LA 70148 USA; 2 Computer-Chemistry-Center, Department of Chemistry and Pharmacy, Friedrich-Alexander-University Erlangen-Nuernberg, Nägelsbachstr. 25, 91052 Erlangen, Germany

**Keywords:** Anion-anion complexes, Ab initio calculations, Charge-dipole interactions, Counter-intuitive bonding

## Abstract

**Background:**

At short range, charge-dipole interactions, which scale with *R*^*−2*^, can override the more conceptually familiar charge-charge ones, which scale with *R*^*−1*^. This can lead to metastable minima for complexes between ions of like charge, but also be designed to create, as shown here, thermodynamically stable anion-anion complexes. We show that such complexes can be stable to dissociation into their component anions, and to autoionization.

**Methods:**

Geometries were optimized at the MP2/aug-cc-pVTZ level, and optimized structures confirmed to be minima or transition states by calculating the normal vibrations within the harmonic approximation at this level. Energies were refined using these geometries for CCSD(T) calculations with the same basis set. MP2/aug-cc-pVTZ single points with the complexed chloride ion represented by a point charge were used to demonstrate the charge-dipole nature of the bonding interaction. All calculations used Gaussian16.

**Supplementary Information:**

The online version contains supplementary material available at https://doi.org/10.1007/s00894-026-06947-6.

## Introduction

“Electrostatic interactions” in chemistry are very often understood to represent only charge-charge (monopole-monopole) interactions, which obey Coulomb’s law and scale with *R*^*−1*^, where *R* is the effective distance between the charge centers in point-charge simplifications of inter-fragment interactions. However, at short range, charge-dipole interactions, which scale with *R*^*−2*^, become important, and can even lead to metastable minima in complexes between anions [1]. Anion-anion dimers and other architectures involving periodates, iodates and bromates have also been reported recently [2, 3]. Placing the emphasis on charge-charge interactions, which are of course repulsive between ions of like charge, has led to the concept of “anti-electrostatic” bonds [4, 5], although it is generally accepted that this term is not appropriate [6], and that “counter-intuitive” is a better way of describing these interactions [7–9]. Interest in these types of interactions has been discussed by others earlier [10, 11]. A comprehensive review of anion-anion interactions via σ- and π-holes was published in 2024 [12].

Minimum-energy, usually metastable, complexes between ions of like charge, and especially of dianion complexes, have attracted much interest. It is often mistakenly believed that dianions cannot exist in the gas phase because they would dissociate spontaneously in a “Coulomb explosion,” or lose an electron spontaneously, but Dreuw and Cederbaum summarized the criteria that lead to stable multiply charged gas-phase anions in 2002 [13].

We have now set out to demonstrate how anion-anion complexes can be made thermodynamically stable, and also stable to autoionization in the gas phase, using strong monopole-dipole interactions. The system that we have chosen consists of the poly-yne 1-carboxylate-ω-iodo- anions 1 (*N* = *1–5*) complexed via the terminal iodine to a chloride anion.

**Figure:**
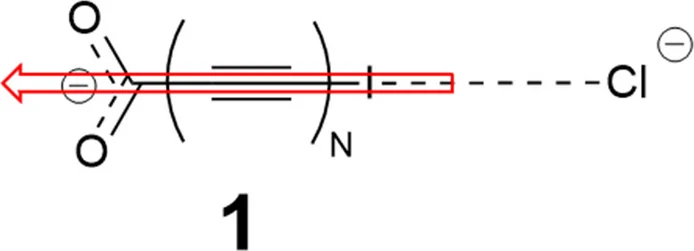

The red arrow indicates the dipole moment of the anion (arrowhead at the negative end). 1(N = 4) has been investigated computationally by Schreiner [14] in search of a bound anion-anion complex. We have now included this compound in a systematic search of the effect of N on the stability of the halogen-bonded complex.

## Calculational methods

All calculations used the aug-cc-pVTZ basis set [15, 16] with the aug-cc-pVTZ-PP pseudopotential basis set for iodine [17, 18] obtained from the Basis Set Exchange [19]. Geometry optimizations and calculations of the normal vibrations within the harmonic approximation used second-order Møller-Plesset (MP2) theory [20–22]. Energies were refined by single-point calculations on the MP2 geometries using the same basis set with CCSD(T) [23]. *V*_S(max)_ values were determined on the 0.001 a.u. isodensity surface using Multiwfn [24].

## Results

### Electrostatic properties of the dipolar anions

**Fig. 1:**
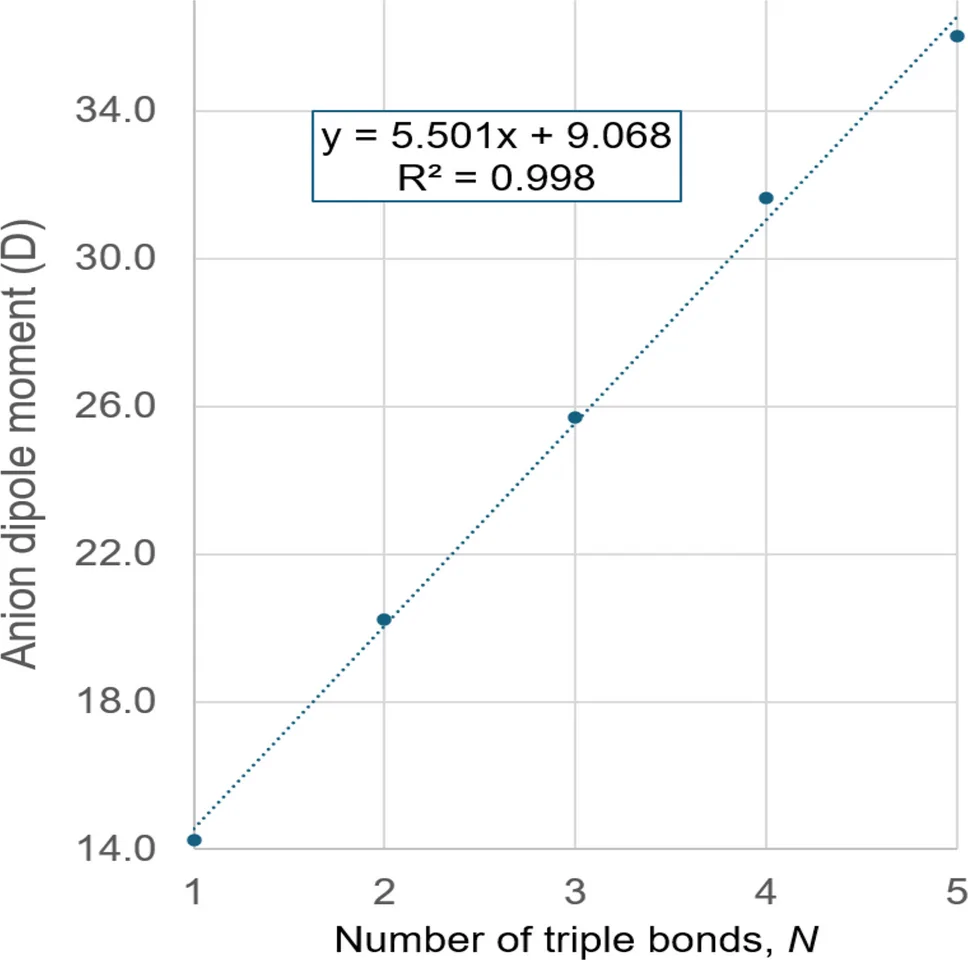
The calculated dipole moment of the anions 1 (MP2/aug-cc-pVTZ) plotted against the number of triple bonds *N*. The dotted line shows the best-fit linear regression line and the inset box shows the regression equation and *R*^*2*^

Because dipole moments for charged species depend on the origin used in their calculation, the dipole moments discussed here use the center of nuclear charge as the origin. Figure 1 shows a plot of the calculated dipole moments (MP2/aug-cc-pVTZ using the MP2 density) for the anions **1** against the number of triple bonds, N.

Table 1 shows details of the calculation results for the five systems.

**Table 1:** The dipole moments of the uncomplexed anions (μ_(anion)_), *V*_S(max)_ for the iodine of these anions, the CCSD(T)//aug-cc-pVTZ + MP2-ZPE energy relative to the separated anions (E_(minimum)_), the iodine-chlorine distances in the minimum-energy complex (R_I-Cl_) and in the dissociation transition state (R^TS^) and the CCSD(T)//aug-cc-pVTZ + MP2-ZPE dissociation barrier for the anion-anion complexes

***N***	μ_(anion)_ (Debye)	*V*_S(max)_ (kcal mol^−1^)	E_(minimum)_ (kcal mol^−1^)	R_I-Cl_ (Å)	μ_(complex)_ (Debye)	R^TS^ (Å)	Barrier (kcal mol^−1^)
1	14.24	−23.66	31.05	3.206	3.50	4.423	1.47
2	20.23	−8.20	18.81	3.017	8.39	5.172	6.17
3	25.69	1.70	10.34	2.934	11.90	5.783	10.13
4	31.64	8.83	1.74	2.884	14.68	6.343	15.58
5	36.04	14.20	−1.53	2.852	16.99	6.912	16.30

**Fig. 2:**
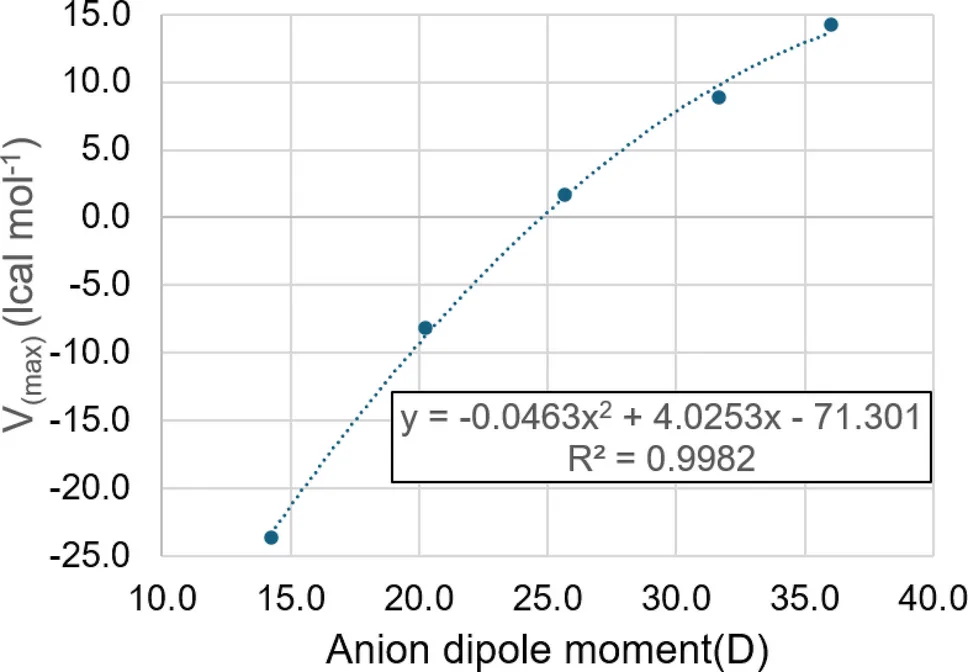
The HF/aug-cc-pVTZ calculated *V*_*S(max*)_ for the σ-hole on the iodine atoms of the anions **1** plotted against the dipole moment of the anion. The dotted line shows the best-fit polynomial (order 2) regression line and the inset box shows the regression equation and *R*^*2*^

We note here that dipole moments for charged species are origin-dependent, which makes comparisons slightly indefinite. Here, we have used the center of nuclear charge of each anion as the origin, so that the trends in dipole moments should be real. As expected, the dipole moment depends linearly on the length of the molecule, which also correlates with *N*. Thus, the series of anions **1** gives us the opportunity to investigate the dependence of the binding energy relative to separated **1** and chloride on the dipole moment. This comparison is, however, not simple because *V*_*S(max)*_, the most positive value of the molecular electrostatic potential (MEP) on the 0.001 a.u. isodensity surface at the center of the σ-hole [25, 26] on iodine, also depends on the dipole moment, so that *V*_*S(max)*_ at the position facing the complexed chloride anion is not constant throughout the series of anions. Figure 2 shows the dependence of *V*_*S(max)*_ on the anion dipole moment. The calculated *V*_*S(max)*_ values are also shown in Table 1.

The figure shows that *V*_*S(max)*_ is positive for *N* ≥ *3*, which is a little surprising for a series of anions, especially as the value for *N* = *5* is almost 15 kcal mol^−1^. It demonstrates, however, the effect of the large dipole moment (for *N* = *5*, 36 Debye) in reinforcing the σ-hole, which without the dipole effect would be less positive.

The correlation is not quite linear but shows a clear trend to more positive *V*_*S(max)*_ as the length of the molecule, and hence the dipole moment, increases. All in all, both the calculated dipole moments of the series of anions and the *V*_*S(max)*_ values of the iodine substituent indicate that complexation of an anion, in our case chloride, at the σ-hole position on the iodine should become less unfavorable as the number of triple bonds increases, and that we can expect metastable minima for the anion-anion complex.

Note that the distances between the center of charge, or the center of nuclear charge used as the origin for the dipoles, and the chloride ion (or the point charge used below) are significantly larger than the estimated 3 Å limit, below which the monopole-dipole approximation is estimated to break down. [27].

### Complexation and dissociation

**Fig. 3:**
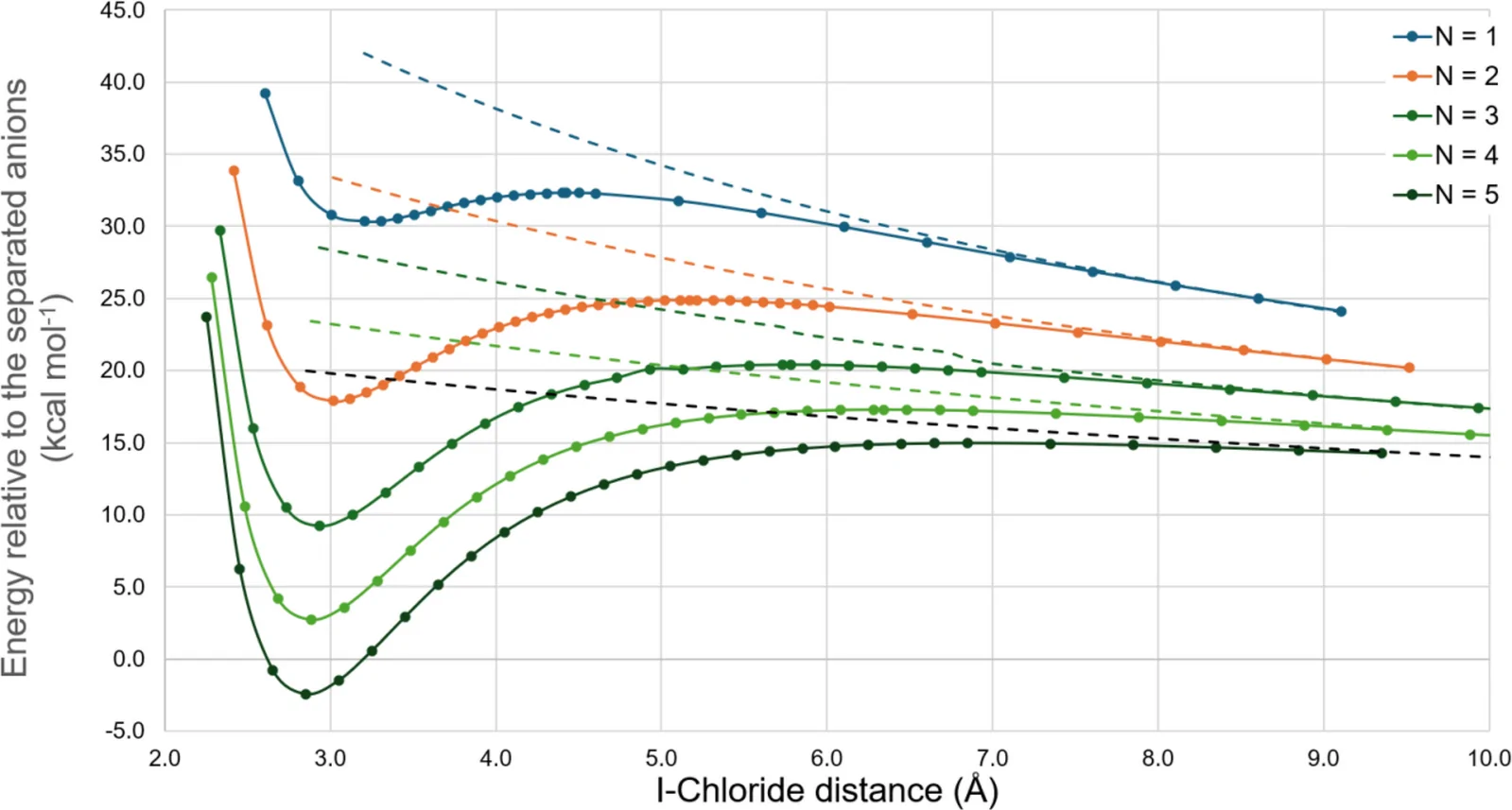
Calculated I − Cl dissociation curves (MP2/aug-cc-pVTZ) for the complexes of anions **1** with a chloride anion. The dashed lines show the estimated Coulomb repulsion curves for the ions in the same color as the dissociation energy profiles (see text)

Figure 3 shows the calculated (MP2/aug-cc-pVTZ) dissociation curves for the anion pairs for *N* = 1–5. All the systems investigated showed minima for the halogen-bonded complex. As expected for charge-dipole complexes, these minima become deeper and the barriers to dissociation larger as the dipole moment of anion **1** increases. The depth of the minima, which for *N* = 1–4 are metastable relative to the separated anions, can be judged by comparing the calculated dissociation curves with the dashed line, which are estimated Coulomb repulsion curves for each of the complexes. These curves were constructed by fitting the repulsion calculated from Coulomb’s Law to the long-range regions of the calculated dissociation energy profiles.

**Fig. 4:**
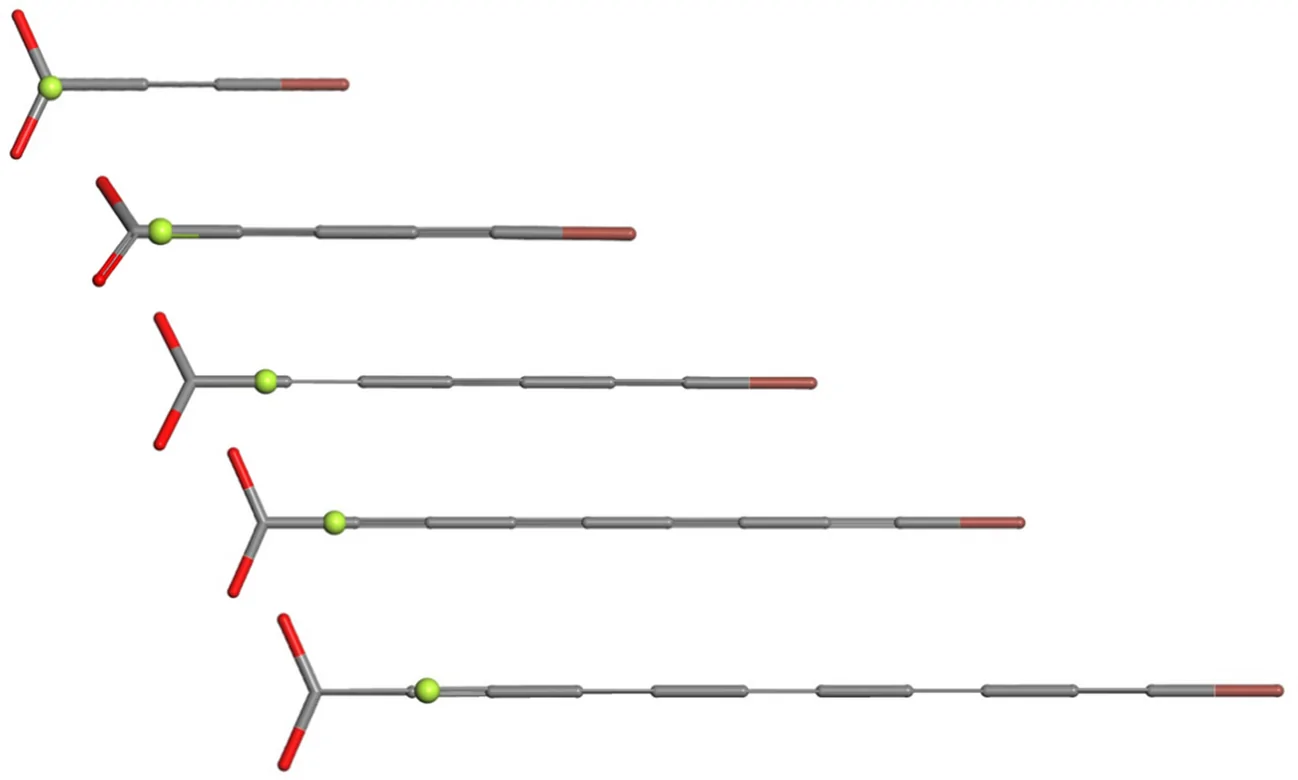
The fitted centers of charge (green spheres) for the five anions **1**

Figure 4 shows the positions of the effective centers of charge for the five uncomplexed dianions, as given by the Coulomb fit to the dissociation curves. profiles. The effective charge center, which need not correspond to the real center of charge because it is fitted to a point-charge model, is centered on the carboxylate carbon for *N* = 1, and moves progressively towards, and past C_2_ with increasing size up to *N* = 5. These results are intuitively reasonable.

For *N* = 5, the anion-anion complex is marginally stable relative to the separated anions, and is therefore an unusual example of a complex between ions of like charge that is bound towards dissociation in the gas phase. The trends show that we can expect anions in this series with more than five triple bonds to be bound clearly. The barriers to dissociation for the metastable minima increase steadily as N increases, but this trend flattens out after *N* = 4.

As expected, the dipole moments of the anion-anion complexes are consistently lower than for the isolated anions, and the difference increases from 10.7 Debye for *N* = 1 to 19.1 for *N* = 5.

**Fig. 5:**
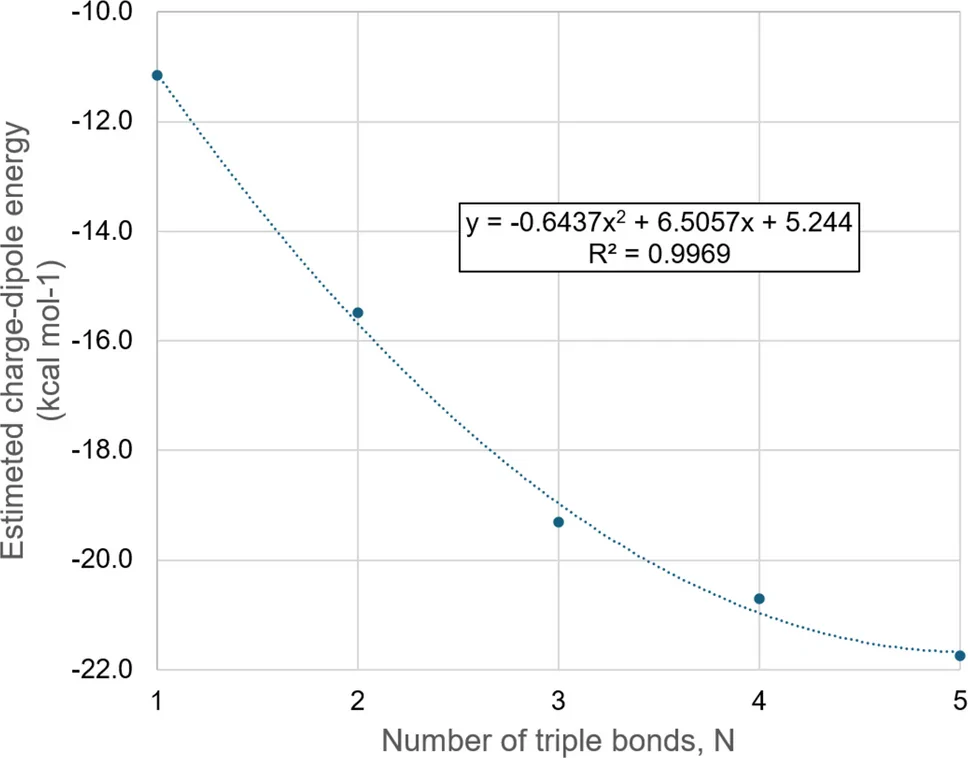
Estimated charge-dipole interaction energies as a function of the number of triple bonds in the anion **1**. The energies are the difference between the calculated minimum energy relative to the separated anions and the estimated charge-charge repulsion energy (dashed lines in Fig. 3)

We can estimate the charge-dipole stabilization as the difference between the energy of the minimum and that of the estimated charge-charge profile (dashed curves in Fig. 3). The resulting energies are shown in Fig. 5.

In order to compare these binding energies with a neutral halogen-bonded equivalent, we calculated the halogen bonded complex 2, in which the poly-yne is a neutral isoelectronic equivalent of the anion 1(N = 5).

**Figure:**
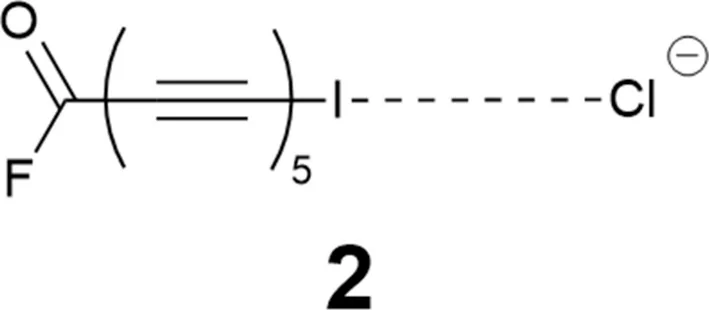

The optimized I…Cl distance in this complex is 2.742 Å, compared to 2.852 in the isoelectronic anion-anion complex. The CCSD(T)/aug-cc-pVTZ binding energy including the MP2/aug-cc-pVTZ zero-point energy is −33.1 kcal mol^−1^, compared with an estimated charge-dipole bonding energy of −21.8 kcal mol^−1^ for the anion-anion complex. *V*_S(max)_ on iodine for 2 is 49.9 kcal mol^−1^, compared with +14.2 for the isoelectronic anion. The calculated dipole moment for 2 is 6.0 Debye (36.0 for the anion).

### Point-charge model

**Fig. 6:**
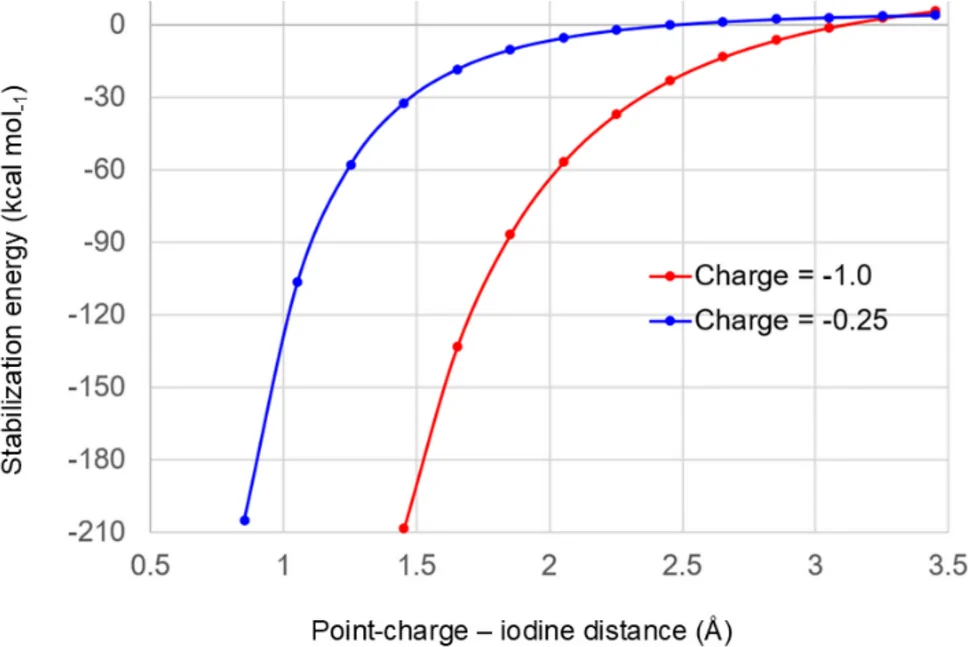
Calculated (MP2/aug-cc-pVTZ) stabilization energies for the interaction of **1(N = 5)** with point charges of magnitude −1.0 and −0.25 a.u. on the linear extension of the C − I bond

Point charge models are established tools for investigating intermolecular interactions [28–33] especially when negative point charges, which cannot act as pseudo-atoms, are used to represent anions. We therefore performed single-point calculations on the optimized geometry of **1(N = 5)** with a point charge on the linear extension of the C − I bond at fixed distances. The results are shown in Fig. 6.

The interaction with the charge of −1 becomes stabilizing at distances shorter than approximately 3.2 Å, and that with the −0.25 charge at approximately 2.4 Å.

**Fig. 7:**
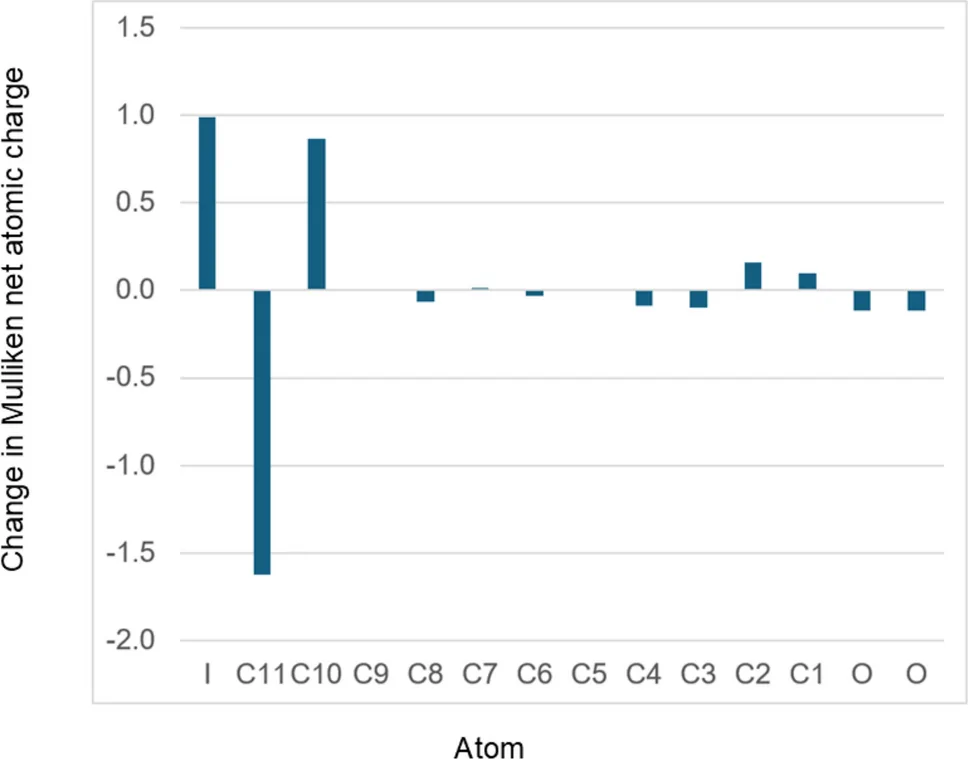
Changes in the Mulliken net atomic charges of 1(N = 5) when polarized by a point charge of − 0.25 collinear with the C − I bond and 1.452 Å from the iodine atom

Surprisingly, the stabilization energy increases as the distance between iodine and the point charge becomes shorter, and no minimum is observed in the range that we investigated. This contrasts with the case investigated previously [1], which gave a shallow metastable minimum with a point charge of −0.5 a.u. Thus, polarization of anion **1** is able to overcome the anion–point charge repulsion at short distances. Figure 7 shows the changes in the Mulliken net atom charges for anion **1(N = 5)** when polarized by a point charge of − 0.25 situated 1.452 Å from the iodine atom.

A full positive charge is induced on the iodine atom, resulting in a strong electrostatic stabilization, despite the negative charge of **1(N = 5)**. This strong polarization serves to strengthen or even replace the stabilization caused by the charge-dipole interaction.

## Summary and conclusions

In principle, “electrostatic” interactions are the simplest to understand in chemistry. However, “electrostatic” is commonly understood as only to consist of simple charge-charge (monopole-monopole) interactions. As shown by the examples discussed here, electrostatics can be far more complex, and hence give rise to far less expected results than suggested by the simple monopole-monopole picture. Although the inadequacy of this picture has been pointed out many times, leading to expressions such as “a fallacy of atomic charges” [34] or “a travesty of bonding theory” [35] the expectations of many chemists for “electrostatics” are limited to a non-polarizable monopole-monopole picture. We have repeatedly pointed out the dangers of neglecting polarization [31, 36–39] and more recently emphasized the dominance of monopole-dipole interactions at short distances [1]. However, “chemical intuition” still seems a long way from a realistic polarizable multipole model.

Clearly, as the length of the poly-yne anion increases, the monopole-monopole repulsion decreases (as shown in Fig. 3), and the σ-hole on iodine becomes more positive (see Fig. 2). These two factors alone can result in a stabilization of the anion-anion complex [14]. Of course, the increasing dipole moment makes the σ-hole more positive. However, the occurrence of local minima on the dissociation energy profiles can only is only possible within a multipole expansion of limited order if at least the monopole-dipole term is included. The occurrence of such minima within a qualitative molecular-orbital rationalization would be attributed to resonance, covalent interactions, or charge transfer, depending on the analysis used.

The invented examples investigated here serve to outline the principles for designing charge-dipole bound anion-anion complexes. For relative synthetic simplicity, the triple bonds in **1** could be replaced by polyphenyl chains to give the necessary distance and dipole moment. One example of metastable anion-dipole complex has been reported [40], although the authors chose to describe it as “anti-electrostatic” on the basis of expectations based on an inadequate monopole-monopole model.

## Supplementary Information

Below is the link to the electronic supplementary material.

ESM1 The Gaussian16 archive entries for the CCSD(T) single-point calculations for all the optimized anions and their chloride complexes (8 pages) (PDF 215 KB)

## Data Availability

The datasets generated during and/or analyzed during the current study are available from the corresponding author on reasonable request.

## References

[1] Clark T (2026) The myth of “anti-electrostatic” bonds. J Comput Chem 14:e70410. 10.1002/jcc.70410 42212542

[2] Calabrese M, Pizzi A, Daolio A, Frontera A, Resnati G (2022) Periodate anions as a halogen bond donor: formation of anion-anion dimers and other adducts. Chem Commun 58:9274–9277. 10.1039/d2cc03191d 35904031

[3] Calabrese M, Pizzi A, Beccaria R, Frontera A, Resnati G (2023) Halogen bonding assembles anion-anion architectures in non-centrosymmetric iodate and bromate crystals. ChemPhysChem 24:e202300298. 10.1002/cphc.202300298 37306232

[4] Weinhold F, Klein RA (2014) Anti-electrostatic hydrogen bonds. Angew Chem Int Ed 53:11214–11217. 10.1002/anie.201405812 25196556

[5] Novák M, Marek R, Foroutan-Nejad C (2017) Anti-electrostatic CH-ion bonding in decorated graphanes. Chem Eur J 23:14,931-14,936. 10.1002/CHEM.201703459 28763124

[6] Frenking G, Caramori GF (2015) No Need for a re-examination of the electrostatic notation of the hydrogen bonding: a comment. Angew Chem Int Ed 9:296–2599. 10.1002/anie.201411374 25651534

[7] Murray JS, Shields ZP-I, Seybold PG, Politzer P (2015) Intuitive and counterintuitive interactions of aromatic π regions with the hydrogen and nitrogen of HCN. J Comput Sci 10:209–216. 10.1016/j.jocs.2015.02.001

[8] López-Andarias J, Bauzá A, Sakai N, Frontera A, Matile S (2018) Remote control of anion-π catalysis on fullerene-centered catalytic triads. Angew Chem Int Ed 130:11049–11053. 10.1002/anie.201804092 PMC6120490 29806724

[9] Murray JS, Politzer P (2021) Can counter-intuitive halogen bonding be coulombic? ChemPhysChem 22:1201–1207. 10.1002/cphc.202100202 33844430

[10] Wheeler SE, Houk KN (2010) Are anion/π interactions actually a case of simple charge-dipole interactions? J Phys Chem A 114:8658–8664. 10.1021/jp1010549 PMC3049731 20433187

[11] Geronimo I, Singh NJ, Kim KS (2011) Can electron-rich π systems bind anions? J Chem Theory Comput 7:825–829. 10.1021/ct100686e 26606334

[12] Pizzi A, Dhaka A, Beccaria R, Resnati G (2024) Anion⋯anion self-assembly under the control of σ- and π-hole bonds. Chem Soc Rev 53:6654–6674. 10.1039/d3cs00479a 38867604

[13] Dreuw A, Cederbaum L (2002) Multiply charged anions in the gas phase. Chem Rev 102:181–200. 10.1021/cr0104227 11782132

[14] Scheiner S (2022) Search for an exothermic halogen bond between anions. Phys Chem Chem Phys 24:6964–6972 10.1039/d1cp05628j 35254353

[15] Dunning TH Jr (1989) Gaussian basis sets for use in correlated molecular calculations. I. The atoms boron through neon and hydrogen. J Chem Phys 90:1007–1023. 10.1063/1.456153

[16] Woon DE, Dunning TH Jr (1993) Gaussian-basis sets for use in correlated molecular calculations. 3. The atoms aluminum through argon. J Chem Phys 98:1358–1371. 10.1063/1.464303

[17] Peterson KA, Figgen D, Goll D, Stoll E, Hermann H, Dolg M, (2003) Systematically convergent basis sets with relativistic pseudopotentials. II. Small-core pseudopotentials and correlation consistent basis sets for the post-d group 16–18 elements. J Chem Phys 119:11,113–11,123. 10.1063/1.1622924

[18] Peterson KA, Shepler BC, Figgen D, Stoll H (2006) On the spectroscopic and thermochemical properties of ClO, BrO, IO, and their anions. J Phys Chem A 110:13,877-13,883. 10.1021/jp065887l 17181347

[19] Pritchard BP, Altarawy D, Didier B, Gibson TD, Windus TL (2019) A new basis set exchange: an open, up-to-date resource for the molecular sciences Community. J Chem Inf Model 59:4814–4820. 10.1021/acs.jcim.9b00725 31600445

[20] Head-Gordon M, Pople JA, Frisch MJ (1988) MP2 energy evaluation by direct methods. Chem Phys Lett 153:503–506. 10.1016/0009-2614(88)85250-3

[21] Frisch MJ, Head-Gordon M, Pople JA (1990) Direct MP2 gradient method. Chem Phys Lett 166:275–280. 10.1016/0009-2614(90)80029-D

[22] Head-Gordon M, Head-Gordon T (1994) Analytic MP2 frequencies without fifth order storage: theory and application to bifurcated hydrogen bonds in the water hexamer. Chem Phys Lett 220:122–128. 10.1016/0009-2614(94)00116-2

[23] Purvis GD III, Bartlett RJ (1982) A full coupled-cluster singles and doubles model—the inclusion of disconnected triples. J Chem Phys 76:1910–1918. 10.1063/1.443164

[24] Lu T, Chen F (2012) Multiwfn: a multifunctional wavefunction analyzer. J Comput Chem 33:580–592. 10.1002/jcc.22885 22162017

[25] Clark T, Hennemann M, Murray JS, Politzer P (2007) Halogen bonding: the σ-hole. J Mol Model 13:291–296. 10.1007/s00894-006-0130-2 16927107

[26] Clark T (2013) σ-Holes, WIREs. Comput Mol Sci 3:13–20. 10.1002/wcms.1113

[27] Lacava F (2016) Multipole expansion of the electrostatic potential. In Classical electrodynamics: from image charges to the photon mass and magnetic monopoles. Springer, Cham, pp 17–31. 10.1007/978-3-319-39474-9_2

[28] Hermansson K (2002) Blue-shifting hydrogen bonds. J Phys Chem A 106:4695–4702. 10.1021/jp0143948

[29] Pejov L, Hermansson K (2003) On the nature of blue-shifting hydrogen bonds: ab initio and density functional studies of several fluoroform complexes. J Chem Phys 119:313–324. 10.1063/1.1571517

[30] Hennemann M, Murray JS, Politzer P, Clark T (2011) Polarization-induced σ-holes and hydrogen bonding. J Mol Model 18:2461–2469. 10.1007/s00894-011-1263-5 22015592

[31] Clark T, Murray JS, Politzer P (2014) the role of polarization in a halogen bond. Aus J Chem 67:451–456. 10.1071/CH13531

[32] Clark T, Murray JS, Politzer P (2018) The σ-hole Coulombic interpretation of trihalide anion formation. ChemPhysChem 19:3044–3049. 10.1002/cphc.201800750 30156047

[33] Brinck T, Nyberg Borrfors A (2019) Electrostatics and polarization determine the strength of the halogen bond: a red card for charge transfer. J Mol Model 25:125. 10.1007/s00894-019-4014-7 31020416

[34] Politzer P, Murray JS, Concha MC (2008) σ-hole bonding between like atoms: a fallacy of atomic charges. J Mol Model 14:659–665. 10.1007/s00894-008-0280-5 18317819

[35] Price SL (1996) Applications of realistic electrostatic modelling to molecules in complexes, solids and proteins. J Chem Soc Farad Trans 92:2997–3008. 10.1039/FT9969202997

[36] Hennemann M, Murray JS, Politzer P, Riley KE, Clark T (2012) Polarization induced σ-holes and hydrogen bonding. J Mol Model 18:2461–2469. 10.1007/s00894-011-1263-5 22015592

[37] Clark T, Politzer P, Murray JS (2015) Correct electrostatic treatment of non-covalent interactions: the importance of polarisation. WIREs Comput Mol Sci 5:169–177. 10.1002/wcms.1210

[38] Clark T (2017) Polarization, donor–acceptor interactions and covalent contributions in weak interactions: a clarification. J Mol Model 23:297. 10.1007/s00894-017-3473-y 28956190

[39] Politzer P, Murray JS, Clark T (2019) Explicit inclusion of polarizing electric fields in σ- and π-hole interactions. J Phys Chem A 123(10):123–10130. 10.1021/acs.jpca.9b08750 31647237

[40] Holthoff JM, Engelage E, Weiss R, Huber SM (2020) “Anti-electrostatic” halogen bonding. Angew Chem Int Ed 59(11):150–11157. 10.1002/anie.202003083 PMC7317790 32227661

