# Supplementary material for: Charge-dipole bound anion-anion complexes
Source: J Mol Model. 2026 Sep 15;32(10):369. doi: 10.1007/s00894-026-06947-6 (PMC13578066; doi:10.1007/s00894-026-06947-6)
Supplement: Supplementary file 1 [file 894_2026_6947_MOESM1_ESM.pdf]

Charge-dipole bound anion-anion complexes

Jane S. Murray[a] and Timothy Clark[b]*

[a] Department of Chemistry, LSU New Orleans, New Orleans, LA 70148, USA

[b] Computer-Chemistry-Center, Department of Chemistry and Pharmacy, Friedrich-Alexander-University Erlangen-Nuernberg, Nägelsbachstr. 25, 91052 Erlangen, Germany.

Supplementary Information

**1**	Gaussian archive entries for the Poly-yne 1-carboxylate-ω-iodo- anions **1**	S2
**2**	Gaussian archive entries for the Poly-yne 1-carboxylate-ω-iodo- anion : Chloride complexes	S4
**3**	Gaussian archive entries for the Poly-yne 1-carboxylate-ω-halide-anions : and their Chloride complexes	S6
**4**	Gaussian archive entries for the neutral analog 2 : and its Chloride complex	S8

1. Gaussian archive entries for the Poly-yne 1-carboxylate-ω-iodo- anions 1

1.1. N=1

```
1\1\FAU-CCC-CCQC155\SP\RCCSD(T)-FC\GenECP\C3I1O2(1-)\CLARK\28-May-2026
\0\\# ccsd(t)/genecp guess=read geom=check\\O2-CC-I...Cl-\\-1,1\C,0,-3
.8268979989,0.6877565295,-0.0053794013\C,0,-2.3180902083,0.6663300394,
0.0068176668\C,0,-1.0907464642,0.6488884607,0.0157389624\I,0,0.8916662
776,0.6206302753,0.0317624235\O,0,-4.3246326124,1.8366475401,-0.000101
8112\O,0,-4.3568039937,-0.446605845,-0.0186438402\\Version=ES64L-G16Re
vA.03\State=1-A\HF=-558.0564201\MP2=-559.2191087\MP3=-559.2014612\MP4D
=-559.2314077\MP4DQ=-559.1995008\MP4SDQ=-559.2174632\CCSD=-559.2112733
\CCSD(T)=-559.2733953\RMSD=4.086e-09\PG=C01 [X(C3I1O2)]\\@
```

1.2 N=2

```
1\1\FAU-CCC-CCQC157\SP\RCCSD(T)-FC\GenECP\C5I1O2(1-)\CLARK\28-May-2026
 \0\\# ccsd(t)/genecp guess=read geom=check\\O2-CC-I...Cl-\\-1,1\C,0,-2
 .943470555,0.6873278155,0.0033949439\C,0,-1.4384645335,0.6832431386,0.
 0117478479\C,0,-0.2044930333,0.6804870866,0.0168142965\I,0,4.358851861
 4,0.6723037667,0.0544228678\O,0,-3.4500921024,1.8316651999,-0.00050892
 02\O,0,-3.4562960882,-0.454223938,0.0015456985\C,0,2.3838879512,0.6752
 28807,0.0252443894\C,0,1.1523004997,0.6777221237,0.0245948761\\Version
 =ES64L-G16RevA.03\State=1-A\HF=-633.760003\MP2=-635.2251321\MP3=-635.1
 982411\MP4D=-635.2391565\MP4DQ=-635.1939506\MP4SDQ=-635.2167668\CCSD=-
 635.2093942\CCSD(T)=-635.2929287\RMSD=1.662e-09\PG=C01 [X(C5I1O2)]\\@
```

1.3 N=3

```
1\1\FAU-CCC-CCQC158\SP\RCCSD(T)-FC\GenECP\C7I1O2(1-)\CLARK\28-May-2026
 \0\\# ccsd(t)/genecp guess=read geom=check\\O2-CCCCCC-I...Cl-\\-1,1\C,
 0,-1.0457208192,0.7014398301,0.0052210011\C,0,0.4599200616,0.710669261
 6,0.0112499404\C,0,1.6967420308,0.7182887742,0.0161321786\I,0,8.840230
 2917,0.7640806987,0.0546814027\O,0,-1.5584472983,1.8420082958,0.001256
 4604\O,0,-1.5444343704,-0.4453399395,0.0051255197\C,0,6.867204704,0.75
 1308706,0.0396051513\C,0,5.6335095624,0.7433691666,0.0359114641\C,0,3.
 0441778271,0.7266902123,0.0217952246\C,0,4.2845050102,0.7347209942,0.0
 29170657\\Version=ES64L-G16RevA.03\State=1-A\HF=-709.4620453\MP2=-711.
 2317958\MP3=-711.1939719\MP4D=-711.2465462\MP4DQ=-711.1868621\MP4SDQ=-
 711.2144882\CCSD=-711.2063196\CCSD(T)=-711.3120587\RMSD=1.084e-09\PG=C
 01 [X(C7I1O2)]\\@
```

1.4 N=4

```
1\1\FAU-CCC-CCQC156\SP\RCCSD(T)-FC\GenECP\C9I1O2(1-)\CLARK\30-May-2026
 \0\\# ccsd(t)/genecp guess=read geom=check\\O2-CCCCCCCC-I\\-1,1\C,0,-7
 .6484748558,-0.0001901097,-0.0011097005\C,0,-6.1419577052,-0.000153133
 1,-0.0001306135\C,0,-4.9036483219,-0.0000148378,0.0006400428\I,0,4.818
 1836367,-0.0004025499,0.0088309549\O,0,-8.1513000812,-1.1443750146,-0.
 001458604\O,0,-8.1513557473,1.143969856,-0.0014121138\C,0,2.8465446053
 ,-0.000063721,0.0069667476\C,0,1.6119374696,0.0000805567,0.0058465001\
 C,0,-3.5605331812,-0.0000737756,0.0016422683\C,0,-2.3162975852,0.00070
 08348,0.0022502584\C,0,-0.9777836816,-0.0000829406,0.0037593116\C,0,0.
 2657104478,0.0005438347,0.004570948\\Version=ES64L-G16RevA.03\State=1-
 A\HF=-785.1622325\MP2=-787.238151\MP3=-787.1882132\MP4D=-787.2530262\M
 P4DQ=-787.178136\MP4SDQ=-787.2105346\CCSD=-787.2017868\CCSD(T)=-787.33
 01132\RMSD=2.142e-09\PG=C01 [X(C9I1O2)]\\@
```

1.5 N=5


```
1\1\FAU-CCC-CCQC159\SP\RCCSD(T)-FC\GenECP\C11I1O2(1-)\CLARK\30-May-202
 6\0\\# ccsd(t)/genecp guess=read geom=check\\O2-CCCCCCCCCCC-I\\-1,1\C,0
 ,1.0357039793,0.675889729,0.054230654\C,0,2.5428910934,0.6800445652,0.
 0824168992\C,0,3.7816519452,0.6832564282,0.1050268199\I,0,16.077364667
 2,0.6982476706,0.3689065129\O,0,0.5318410805,1.8189784413,0.0457249808
 \O,0,0.538281536,-0.4700058174,0.0439147621\C,0,14.1074762993,0.697207
 6525,0.3163029581\C,0,12.8729145035,0.6961028776,0.2833549839\C,0,5.12
 21282144,0.6859805802,0.1290584559\C,0,6.3680110741,0.6884831752,0.151
 8463996\C,0,10.2841130729,0.6935874258,0.2209697028\C,0,11.5286105291,
 0.6950108027,0.247863657\C,0,7.7014500338,0.6904326622,0.1728405441\C,
 0,8.9492199713,0.6921078069,0.1935486696\\Version=ES64L-G16RevA.03\Sta
 te=1-A\HF=-860.8610312\MP2=-863.2441146\MP3=-863.1812692\MP4D=-863.258
 7203\MP4DQ=-863.1681112\MP4SDQ=-863.2052713\CCSD=-863.1961209\CCSD(T)=
 -863.3472669\RMSD=2.611e-09\PG=C01 [X(C11I1O2)]\\@
```

2. Gaussian archive entries for the Poly-yne 1-carboxylate-ω-iodo- anion : Chloride complexes

2.1. N = 1

```
1\1\FAU-CCC-CCQC156\SP\RCCSD(T)-FC\GenECP\C3Cl1I1O2(2-)\CLARK\28-May-2
 026\0\\# ccsd(t)/genecp guess=read geom=check\\O2-CC-I...Cl-\\-2,1\C,0
 ,-3.7984015889,0.6848870368,-0.0149255298\C,0,-2.2996758995,0.66858097
 72,0.0167445073\C,0,-1.0690418541,0.6552828331,0.0425864019\I,0,0.9518
 567439,0.6333450778,0.0851264841\Cl,0,4.1566645229,0.5985618478,0.1526
 310889\O,0,-4.3253972504,1.8280687604,-0.0381243327\O,0,-4.350601674,-
 0.4465825331,-0.0145356196\\Version=ES64L-G16RevA.03\State=1-A\HF=-101
 7.5739629\MP2=-1018.9517078\MP3=-1018.9492344\MP4D=-1018.9832406\MP4DQ
 =-1018.9447532\MP4SDQ=-1018.9633089\CCSD=-1018.9567965\CCSD(T)=-1019.0
 295765\RMSD=1.278e-09\PG=C01 [X(C3Cl1I1O2)]\\@
```

2.2. N=2

```
1\1\FAU-CCC-CCQC156\SP\RCCSD(T)-FC\GenECP\C5Cl1I1O2(2-)\CLARK\28-May-2
 026\0\\# ccsd(t)/genecp guess=read geom=check\\O2-CC-I...Cl-\\-2,1\C,0
 ,-2.9401103627,0.6931508444,-0.0074762377\C,0,-1.4412856539,0.68499824
 04,0.010158672\C,0,-0.2074561343,0.6783004824,0.025732856\I,0,4.421381
 3227,0.6529835588,0.0848258006\Cl,0,7.4385364177,0.6365413323,0.124819
 7312\O,0,-3.46431711,1.8352207691,-0.0213925607\O,0,-3.4768872621,-0.4
 43150013,-0.0061224867\C,0,2.3944077894,0.6641255714,0.0580045887\C,0,
 1.1581749932,0.6709002143,0.0422006367\\Version=ES64L-G16RevA.03\State
 =1-A\HF=-1093.2951933\MP2=-1094.9774116\MP3=-1094.9654003\MP4D=-1095.0
 103824\MP4DQ=-1094.9584719\MP4SDQ=-1094.9820543\CCSD=-1094.9743037\CCS
 D(T)=-1095.0688418\RMSD=2.138e-09\PG=C01 [X(C5Cl1I1O2)]\\@
```

2.3. N=3

```
1\1\FAU-CCC-CCQC155\SP\RCCSD(T)-FC\GenECP\C7Cl1I1O2(2-)\CLARK\29-May-2
 026\0\\# ccsd(t)/genecp guess=read geom=check\\O2-CCCCCC-I...Cl-\\-2,1
 \C,0,-1.0452907501,0.7083119241,-0.0039611546\C,0,0.4559842356,0.71370
 35601,0.008431251\C,0,1.6915715799,0.7181922052,0.0187061798\I,0,8.915
 2213818,0.7452262757,0.0804743252\Cl,0,11.8487752797,0.75644137,0.1096
 06011\O,0,-1.5721515894,1.8469005645,-0.0228221438\O,0,-1.5642236996,-
 0.4340174823,0.0062751688\C,0,6.8834437863,0.7374606428,0.0593127931\C
 ,0,5.6437661311,0.7328176917,0.0512961172\C,0,3.0481046812,0.723176307
 3,0.029996102\C,0,4.2884399634,0.7278419408,0.0406203502\\Version=ES64
 L-G16RevA.03\State=1-A\HF=-1169.0103136\MP2=-1170.997887\MP3=-1170.975
 0093\MP4D=-1171.0315951\MP4DQ=-1170.9652985\MP4SDQ=-1170.9937907\CCSD=
 -1170.9851212\CCSD(T)=-1171.1019616\RMSD=1.713e-09\PG=C01 [X(C7Cl1I1O2
 )]\\@
```

2.4. N=4

```
1\1\FAU-CCC-CCQC157\SP\RCCSD(T)-FC\GenECP\C9Cl1I1O2(2-)\CLARK\29-May-2
 026\0\\# ccsd(t)/genecp guess=read geom=check\\O2-CCCCCCCC-I...Cl-\\-2
 ,1\C,0,-0.1789402185,0.702945267,0.0065353787\C,0,1.3233541365,0.69807
 71165,0.0557772462\C,0,2.5592897308,0.6942817064,0.0959277892\I,0,12.3
 65392679,0.6625301025,0.4052062351\Cl,0,15.2481391633,0.6513119343,0.5
 02184266\O,0,-0.6951397465,1.8428695827,0.0756423741\O,0,-0.6969422413
 ,-0.4336083243,-0.096443111\C,0,10.3314526144,0.6702777526,0.337447370
 8\C,0,9.0900472501,0.6742715546,0.3014908888\C,0,3.9104656647,0.689863
 6122,0.140695374\C,0,5.1527486705,0.6859148138,0.1801829882\C,0,6.4976
 368276,0.6818164169,0.2219431278\C,0,7.7412354694,0.678355465,0.260751
 0721\\Version=ES64L-G16RevA.03\State=1-A\HF=-1244.7204953\MP2=-1247.01
 4637\MP3=-1246.9796999\MP4D=-1247.0484666\MP4DQ=-1246.9670788\MP4SDQ=-
 1247.0004545\CCSD=-1246.9910571\CCSD(T)=-1247.1305702\RMSD=3.183e-09\P
 G=C01 [X(C9Cl1I1O2)]\\@
```

2.5. N=5


```
1\1\FAU-CCC-CCQC157\SP\RCCSD(T)-FC\GenECP\C9Cl1I1O2(2-)\CLARK\29-May-2
 026\0\\# ccsd(t)/genecp guess=read geom=check\\O2-CCCCCCCC-I...Cl-\\-2
 ,1\C,0,-0.1789402185,0.702945267,0.0065353787\C,0,1.3233541365,0.69807
 71165,0.0557772462\C,0,2.5592897308,0.6942817064,0.0959277892\I,0,12.3
 65392679,0.6625301025,0.4052062351\Cl,0,15.2481391633,0.6513119343,0.5
 02184266\O,0,-0.6951397465,1.8428695827,0.0756423741\O,0,-0.6969422413
 ,-0.4336083243,-0.096443111\C,0,10.3314526144,0.6702777526,0.337447370
 8\C,0,9.0900472501,0.6742715546,0.3014908888\C,0,3.9104656647,0.689863
 6122,0.140695374\C,0,5.1527486705,0.6859148138,0.1801829882\C,0,6.4976
 368276,0.6818164169,0.2219431278\C,0,7.7412354694,0.678355465,0.260751
 0721\\Version=ES64L-G16RevA.03\State=1-A\HF=-1244.7204953\MP2=-1247.01
 4637\MP3=-1246.9796999\MP4D=-1247.0484666\MP4DQ=-1246.9670788\MP4SDQ=-
 1247.0004545\CCSD=-1246.9910571\CCSD(T)=-1247.1305702\RMSD=3.183e-09\P
 G=C01 [X(C9Cl1I1O2)]\\@
```

3. Gaussian archive entries for the Poly-yne 1-carboxylate-ω-halide-anions : and their Chloride complexes

3.1. N=5, X=Br, Anion

1\1\FAU-CCC-CCQC155\SP\RCCSD(T)-FC\Gen\C11Br1O2(1-)\CLARK\01-Jun-2026\
0\\# ccsd(t)/gen guess=read geom=check\\O2-CCCCCCCCCC-Br\\-1,1\C,0,1.0
496538031,0.6770519018,0.0495091769\C,0,2.5568449566,0.6796350419,0.07
99360186\C,0,3.7955437461,0.6817609322,0.1045771693\Br,0,15.8949151383
,0.7019534278,0.3436693677\O,0,0.5471688226,1.8206053714,0.0282500943\
O,0,0.5506537877,-0.4682216068,0.0504640117\C,0,14.1197796314,0.698961
84,0.3083600291\C,0,12.8889556316,0.6968775007,0.2838341335\C,0,5.1361
769548,0.6841088632,0.131606513\C,0,6.381821801,0.6863187047,0.1562341
803\C,0,10.2990700378,0.6926321505,0.233187097\C,0,11.5424473104,0.694
5983127,0.2570510285\C,0,7.7157202005,0.6886028993,0.1828863797\C,0,8.
962906178,0.6904386605,0.2064408005\\Version=ES64L-G16RevA.03\State=1-
A\HF=-3138.6586987\MP2=-3141.0575677\MP3=-3140.9972832\MP4D=-3141.0738
481\MP4DQ=-3140.983454\MP4SDQ=-3141.020724\CCSD=-3141.0115222\CCSD(T)=
-3141.1628546\RMSD=1.373e-09\PG=C01 [X(C11Br1O2)]\\@

3.2. N=5, X=Br, Anion:Chloride complex

1\1\FAU-CCC-CCQC158\SP\RCCSD(T)-FC\Gen\C11Br1Cl1O2(2-)\CLARK\04-Jun-20
26\0\\# ccsd(t)/gen Guess=read geom=check\\O2-CCCCCCCCCC-I...Cl-\\-2,1
\C,0,1.0585314632,0.6962914653,0.0419284601\C,0,2.5626832869,0.6915459
872,0.0746692168\C,0,3.7996161994,0.6876010396,0.1017704061\Br,0,15.94
967825,0.6485577727,0.3744816037\Cl,0,18.8241635177,0.6388665786,0.442
6760089\O,0,0.5502872545,1.8398963015,0.0029287517\O,0,0.5418514566,-0
.4440703419,0.0586191863\C,0,14.1481714516,0.6544926509,0.3323295049\C
,0,12.9100848146,0.6585982055,0.3032905515\C,0,5.1478285202,0.68332260
46,0.1304189296\C,0,6.3916337571,0.6794169894,0.1569873932\C,0,10.3191
290879,0.66684184,0.2445790629\C,0,11.5640257683,0.6628688252,0.271513
3151\C,0,7.7324615119,0.6750802407,0.1875284427\C,0,8.9787216601,0.670
9908408,0.2142501663\\Version=ES64L-G16RevA.03\State=1-A\HF=-3598.2130
03\MP2=-3600.8296217\MP3=-3600.7843987\MP4D=-3600.8648888\MP4DQ=-3600.
7681541\MP4SDQ=-3600.8063479\CCSD=-3600.7964379\CCSD(T)=-3600.9587518\
RMSD=2.143e-09\PG=C01 [X(C11Br1Cl1O2)]\\@

3.3. N=5, X=Cl, Anion

1\1\FAU-CCC-CCQC155\SP\RCCSD(T)-FC\Gen\C11Cl1O2(1-)\CLARK\02-Jun-2026\
0\\# ccsd(t)/gen guess=read geom=check\\O2-CCCCCCCCCC-Br\\-1,1\C,0,1.0
588841965,0.6771662121,0.0546495806\C,0,2.5666922179,0.6796280574,0.07
90968216\C,0,3.8054491067,0.6817167027,0.102072857\Cl,0,15.7636108536,
0.7018990285,0.3535918644\O,0,0.5563273087,1.8207927246,0.0469119739\O
,0,0.5600457584,-0.4680736874,0.0464846284\C,0,14.1293841676,0.6991518
129,0.3146599119\C,0,12.9007814861,0.6967237763,0.2861454119\C,0,5.146
3153749,0.6841385736,0.1263373313\C,0,6.3918948613,0.6862433868,0.1500
798063\C,0,10.3101657918,0.6924443537,0.2276489909\C,0,11.552758053,0.
694588205,0.2532401523\C,0,7.7262274574,0.6884518993,0.1758370932\C,0,
8.9731213661,0.6904529546,0.1992495763\\Version=ES64L-G16RevA.03\State
=1-A\HF=-1025.7146598\MP2=-1028.0698985\MP3=-1028.0197991\MP4D=-1028.0
954308\MP4DQ=-1028.0046271\MP4SDQ=-1028.0404112\CCSD=-1028.0317574\CCS
D(T)=-1028.1820921\RMSD=3.117e-09\PG=C01 [X(C11Cl1O2)]\\@

3.4. N=5, X=Cl, Anion:Chloride complex

1\1\FAU-CCC-CCQC157\SP\RCCSD(T)-FC\Gen\C11Cl2O2(2-)\CLARK\04-Jun-2026\
0\\# ccsd(t)/gen guess=read geom=check\\O2-CCCCCCCCCC-I...Cl-\\-2,1\C,
0,1.0749138757,0.6975473125,0.0388187277\C,0,2.5793779903,0.6924667857
,0.0733913138\C,0,3.8163523642,0.6883131696,0.1014444132\Cl,0,15.79669
91828,0.6475267827,0.3696324673\Cl,0,18.7657876312,0.6376395867,0.4360

528341\O,0,0.5668276293,1.8416659322,0.0387762529\O,0,0.5596487766,-0.
4431175133,0.0153332859\C,0,14.1614249973,0.6530683535,0.3330185659\C,
0,12.9270896033,0.657294489,0.3052997166\C,0,5.1641918182,0.6837743891
,0.1326383728\C,0,6.4079080997,0.6795782139,0.160745954\C,0,10.3358042
662,0.6663620589,0.2483580923\C,0,11.5795070688,0.6620686058,0.2751029
587\C,0,7.7487502035,0.6751035869,0.191188713\C,0,8.9945844926,0.67100
92468,0.2181693319\\Version=ES64L-G16RevA.03\State=1-A\HF=-1485.264523
1\MP2=-1487.8339927\MP3=-1487.79984\MP4D=-1487.8791243\MP4DQ=-1487.782
3143\MP4SDQ=-1487.8186728\CCSD=-1487.8094727\CCSD(T)=-1487.9701318\RMS
D=1.984e-09\PG=C01 [X(C11Cl2O2)]\\@

4. Gaussian archive entries for the neutral analog 2 : and its Chloride complex

4.1. Neutral analog 2, N=5

```
1\1\FAU-CCC-CCQC157\SP\RCCSD(T)-FC\GenECP\C11F1I1O1\CLARK\16-Jun-2026\
 0\\# ccsd(t)/genecp guess=read geom=check\\O2-CCCCCCCCCC-I...Cl-\\0,1\
 C,0,1.1299076404,0.5978045107,0.047275802\C,0,2.5601620095,0.683496870
 8,0.0778384498\C,0,3.7873021398,0.6759324435,0.1030556012\I,0,16.07900
 69941,0.6929874761,0.3570460816\F,0,0.5702522914,1.8264775949,0.032095
 7817\O,0,0.4624413105,-0.3903864041,0.0354136754\C,0,14.1127926332,0.6
 91476834,0.3154619549\C,0,12.878962945,0.690782778,0.2896052281\C,0,5.
 1366174615,0.6801287668,0.1314661921\C,0,6.3744982325,0.6824712164,0.1
 566089451\C,0,10.2937853519,0.6879621269,0.235368547\C,0,11.5350313335
 ,0.6894543047,0.2609205671\C,0,7.7141168423,0.6851794982,0.183181243\C
 ,0,8.9561608141,0.6866039831,0.2083529311\\Version=ES64L-G16RevA.03\St
 ate=1-A\HF=-885.3879355\MP2=-887.7644593\MP3=-887.7106547\MP4D=-887.78
 51981\MP4DQ=-887.6983879\MP4SDQ=-887.732379\CCSD=-887.7243599\CCSD(T)=
 -887.8687846\RMSD=2.473e-09\PG=C01 [X(C11F1I1O1)]\\@
```

4.2. Neutral analog 2, N=5, chloride complex

```
1\1\FAU-CCC-CCQC155\SP\RCCSD(T)-FC\GenECP\C11Cl1F1I1O1(1-)\CLARK\15-Ju
 n-2026\0\\# ccsd(t)/genecp geom=check guess=read\\O2-CCCCCCCCCC-I...Cl
 -\\-1,1\C,0,1.1311489037,0.6177553698,0.0406472631\C,0,2.5541485694,0.
 6946742827,0.071498791\C,0,3.7835193095,0.6769044832,0.1002291266\I,0,
 16.1561305992,0.6585710251,0.3802916042\Cl,0,18.8972199022,0.659564611
 2,0.4423979201\F,0,0.5709496477,1.8547295234,-0.0163063609\O,0,0.43949
 10531,-0.3566183323,0.0587841833\C,0,14.1121192088,0.6583632069,0.3339
 700236\C,0,12.8627766841,0.6588007973,0.3056831646\C,0,5.129408654,0.6
 720146075,0.1316286207\C,0,6.3713232974,0.6683728425,0.159690445\C,0,1
 0.281051622,0.6600923967,0.247190834\C,0,11.531470023,0.6594042947,0.2
 753609363\C,0,7.7048056302,0.6650162547,0.1893807512\C,0,8.9533058956,
 0.6626546365,0.2175246971\\Version=ES64L-G16RevA.03\State=1-A\HF=-1344
 .99668\MP2=-1347.6027764\MP3=-1347.5559366\MP4D=-1347.6369269\MP4DQ=-1
 347.5390964\MP4SDQ=-1347.5766864\CCSD=-1347.5677811\CCSD(T)=-1347.7282
 019\RMSD=1.206e-09\PG=C01 [X(C11Cl1F1I1O1)]\\@
```
